# Longikaurin A, a natural ent-kaurane, suppresses proliferation, invasion and tumorigenicity in oral squamous cell carcinoma cell by via inhibiting PI3K/Akt pathway *in vitro* and *in vivo*

**DOI:** 10.7150/jca.102125

**Published:** 2025-01-01

**Authors:** Yiming Luo, Zixuan Wang, Yufei Li, Linlin Zhang

**Affiliations:** 1 School of Stomatology, Nanjing Medical University, Nanjing 210009, China.; 2 Department of Radiation Oncology, The Affiliated Cancer Hospital of Nanjing Medical University, Jiangsu Cancer Hospital, Nanjing 210009, China.; 3 Postgraduate College, Xuzhou Medical University, Xuzhou 221000, China.; 4 Department of Pharmacology, The Yancheng Clinical College of Xuzhou Medical University, The First people's Hospital of Yancheng, Yancheng, China.

**Keywords:** Longikaurin A, proliferation, invasion, tumorigenicity, PI3K/Akt signaling pathway, oral squamous cell carcinoma

## Abstract

**Background:** Longikaurin A (LK-A), a naturally occurring ent-kaurane diterpenoid, has been identified as a promising anti-cancer agent. This study aims to elucidate the anti-tumorigenic effects of LK-A on oral squamous cell carcinoma (OSCC) cells and to unravel its underlying mechanisms.

**Methods:** *In vitro* assays, including CCK-8 and EdU, were performed to assess cell viability and proliferation. Transwell migration and invasion assays evaluated cell mobility and invasive potential. Apoptotic effects were analyzed using Annexin V-FITC/PI staining and TUNEL assays. Western blot analysis was conducted to examine protein expression related to cell cycle, apoptosis, and the PI3K/Akt signaling pathway. *In vivo* experiments involved treating mouse xenograft models with LK-A and evaluating tumor growth and signaling pathway inhibition through immunohistochemistry and Western blot assays.

**Results:** LK-A significantly suppressed cell viability and proliferation in a dose-dependent manner, with IC50 values of 4.36 μM and 4.93 μM at 24 h, and 1.98 μM and 2.89 μM at 48 h for CAL27 and TCA-8113 cells, respectively. EdU assays revealed a reduction in the EdU positive rate, and cell cycle analysis showed G2/M phase arrest. Western blot analysis confirmed decreased expression of CyclinB1 and Cdc2. LK-A significantly inhibited OSCC cell mobility and invasive potential, with downregulation of MMP-2 and MMP-9 expression. Apoptotic effects were confirmed by increased apoptosis, upregulation of Bax and cleaved caspase-3, and downregulation of Bcl-2. LK-A suppressed the PI3K/AKT signaling pathway, as evidenced by reduced phosphorylation of PI3K, AKT, and mTOR. The AKT activator SC79 reversed the antiproliferative and pro-apoptotic effects of LK-A. *In vivo*, LK-A significantly inhibited tumor growth in mouse xenograft models, with reduced tumor weights and volumes, and no significant loss in body weight. Immunohistochemistry and Western blot assays confirmed the inhibition of p-Akt and Ki-67 expression.

**Conclusion:** These findings suggest that LK-A exerts potent antiproliferative, anti-migratory, and pro-apoptotic effects on OSCC cells through the suppression of the PI3K/AKT signaling pathway, demonstrating its potential as a therapeutic agent for OSCC.

## Introduction

Oral squamous cell carcinoma (OSCC), as a predominant malignancy within the oral cavity, manifests a significant public health concern due to its high incidence and mortality rates globally 1,2. The pathophysiology of OSCC is notably complex, attributed to a myriad of risk factors including tobacco use, alcohol consumption, and viral infections like HPV. The intricate interplay of these factors culminates in genetic and epigenetic alterations that drive the initiation and progression of OSCC 3. Despite substantial progress in diagnostic and therapeutic modalities, the survival rates for OSCC have remained disappointingly stagnant over the last few decades, highlighting a dire need for innovative treatment approaches.

The phosphoinositide 3-kinase/protein kinase B (PI3K/Akt) signaling pathway is central to key cellular processes such as proliferation, survival, and metastasis 4-6. Activation of PI3K leads to the generation of PIP3, which in turn activates Akt. Activated Akt phosphorylates various substrates, including the mammalian target of rapamycin (mTOR), a critical effector in this pathway. mTOR is a kinase that regulates cell growth, proliferation, and survival through its two complexes, mTORC1 and mTORC2. mTORC1 is activated by Akt and promotes protein synthesis, ribosome biogenesis, and nutrient uptake, essential for cell growth and division. mTORC2, involved in the full activation of Akt, also regulates cytoskeletal dynamics, contributing to cell migration and invasion 7,8.

In cancer, including oral squamous cell carcinoma (OSCC), the PI3K/Akt/mTOR pathway is frequently dysregulated 9. This dysregulation can be due to mutations in genes encoding components of the pathway, overexpression of growth factor receptors, or loss of function of tumor suppressor genes like PTEN, leading to enhanced Akt and mTOR activity. The aberrant activation of the PI3K/Akt/mTOR pathway in OSCC promotes tumor growth, survival, and resistance to apoptosis, alongside facilitating angiogenesis and metastasis 10,11. Notably, increased mTOR signaling has been associated with poor prognosis and resistance to therapy in OSCC, making it a significant marker for aggressive tumor behavior and a viable target for therapeutic intervention 12.

Given the critical role of the PI3K/Akt/mTOR pathway in OSCC pathogenesis and its contribution to the aggressive characteristics of the tumor, targeting this pathway offers a strategic approach to cancer therapy. Inhibiting key nodes within the PI3K/Akt/mTOR cascade has emerged as a potential therapeutic strategy to attenuate OSCC progression and improve treatment outcomes.

Longikaurin A (LK-A), a diterpenoid compound derived from the Isodon genus of plants, has garnered attention in the realm of oncology research due to its pronounced anti-cancer properties 13. Studies have indicated that LK-A exerts potent anti-proliferative, pro-apoptotic, and anti-metastatic effects in various cancer models 13-15. Preliminary evidence suggests that its mechanism of action may involve the modulation of key signaling pathways, including PI3K/Akt, however, these interactions remain poorly understood, particularly in the context of OSCC.

In this context, our study aimed to fill the gap in the current understanding of LK-A's anti-cancer effects on OSCC. We hypothesized that LK-A exerts its anti-tumorigenic activities by modulating the PI3K/Akt signaling pathway, leading to suppressed proliferation, invasion, and tumorigenicity of OSCC cells. To test this hypothesis, we employed a comprehensive set of *in vitro* and *in vivo* experiments to investigate the impact of LK-A on OSCC cell lines and xenograft models, focusing on its effects on the PI3K/Akt axis. Furthermore, we explored the potential reversal of these effects by the Akt activator SC79, aiming to delineate the specificity and mechanism of action of LK-A in the context of OSCC. This study not only enhances our understanding of LK-A's pharmacological profile but also contributes to the broader quest for targeted cancer therapies.

## Materials and Methods

### Cell culture and drug treatment

Human OSCC cell lines CAL27 and TCA-8113 were purchased from Fuheng biology (Shanghai, China). All Cells were maintained in recommended culture media supplemented with 10% fetal bovine serum (FBS) and 1% penicillin-streptomycin, in a humidified incubator with 5% CO₂ at 37°C. And the cells were sub-cultured when 90% confluent. LK-A was dissolved in DMSO to prepare stock solutions, and cells were treated with various concentrations for specific time periods.

### CCK-8 assay

The Cell Counting Kit-8 (CCK-8) assay (Dojindo, Cat# CK04) was employed to evaluate cell viability. Cells were treated with 2, 4 and 6 μM LK-A for periods of 24 and 48 h. Following treatment, 10 μL of CCK-8 solution was added to each well. The cells were then incubated with the CCK-8 solution at 37°C for 1 to 4 h. The absorbance of the formazan product, which reflects cell viability, was measured at 450 nm using a microplate reader.

### EdU assay

The EdU (5-ethynyl-2'-deoxyuridine) assay was performed to evaluate the proliferation of OSCC cell lines CAL27 and TCA-8113 after LK-A treatment. CAL27 and TCA-8113 cells were seeded in 96-well plates and allowed to adhere overnight. Cells were treated with varying concentrations of LK-A (0, 2, 4, and 6 μM) for 24 h. After treatment, 10 μM EdU was added to each well, and cells were incubated for an additional 2 h at 37 °C. Cells were then fixed with 4% paraformaldehyde for 15 minutes and permeabilized with 0.5% Triton X-100 in PBS for 20 minutes. EdU incorporation was detected using the Cell-Light EdU Apollo567 *In Vitro* Kit (Guangzhou RiboBio, Cat# C10310-1) according to the manufacturer's instructions. Cells were incubated with the EdU reaction solution containing the Apollo567 for 30 min in the dark, then counterstained with Hoechst 33342 (5 μg/mL) for 10 min to visualize the nuclei. Fluorescent images were captured using an Olympus IX83 fluorescence microscope, and the percentage of EdU-positive cells was calculated by counting the number of EdU-positive cells relative to the total number of Hoechst-stained nuclei in at least three random fields per well.

### Apoptosis analysis using annexin V-FITC/PI staining

After treating OSCC cells with LK-A for the designated time, cells were harvested by 0.25% trypsinization and centrifugation at 300 × g for 5 min. The cell pellet was resuspended in a binding buffer. Each sample of cells was then stained with Annexin V-FITC and propidium iodide (PI) according to the manufacturer's protocol (KeyGEN Biotech, Nanjing, China). The staining reaction was allowed to proceed for 20 min in the dark at room temperature to prevent photobleaching of the fluorochromes. Then the stained cells were assessed by FACSan flow cytometry (BD Biosciences, USA).

### Cell cycle analysis

Cells treated with LK-A were collected, washed with phosphate-buffered saline (PBS), and then fixed with 70% cold ethanol, which preserves the cells for DNA staining. The fixed cells were washed again with PBS to remove ethanol and then stained with PI, which binds stoichiometrically to double-stranded DNA. the cells were incubated with RNase A (0.1 mg/ml), and stained with propidium iodide (PI, Sigma, USA) for 30 min to allow for adequate staining of the DNA. The DNA content of cells was analyzed by flow cytometry (BD Biosciences, USA).

### Western blot analysis

Harvest OSCC cells treated with LK-A and lyse in ice-cold lysis buffer. Cell lysates were prepared using lysis buffer containing protease and phosphatase inhibitors and quantified via BCA assay. Proteins were separated on SDS-PAGE gels and transferred to PVDF membranes, which were then blocked with 5% BSA in TBST. The membranes were incubated overnight at 4°C with primary antibodies against CyclinB1, Cdc2, MMP-2, MMP-9, cleaved caspase-3 (C.casp.-3), caspase-3, caspase-8, Bax, Cytochrome C (Cyt-C), Bcl-2, p-PI3K, p-AKT, AKT, p-mTOR and mTOR, followed by HRP-conjugated secondary antibodies. All primary antibodies were purchased from Wuhan Sanying, Hubei, P.R.C except p-PI3K (Cell Signaling Technology, Inc., Danvers, MA, USA). Detection was achieved using enhanced chemiluminescence and imaged on a chemiluminescent detection system (GE Healthcare, Chicago, USA).

### *In vivo* tumor xenograft model

In the *in vivo* tumor xenograft model, OSCC cells were first cultured and then harvested to prepare a cell suspension for inoculation. Nude mice, typically 6-8 weeks old, were subcutaneously injected with 1 × 10^6 of cells in a volume of 100 μL at the flank region, to establish solid tumors. Following tumor establishment, when tumors reached a measurable size (approximately 100 mm³), the mice were randomized into treatment groups to receive LK-A or a combination thereof, administered intraperitoneally at predetermined doses of LK-A every three days. Tumor growth was monitored regularly by measuring the tumor dimensions with calipers, and tumor volume was calculated using the formula (length × width²)/2. At the end of the study, mice were euthanized, and tumors were excised for subsequent histological and molecular analyses to assess the effects of the treatments on tumor proliferation, apoptosis, and signaling pathway alterations. The study was carried out under protocols approved by the Institutional Animal Care and Use Committee at Jiangsu Medical Vocational College (XMLL-2024-799).

### Statistical analysis

Statistical analysis was performed using software such as SPSS or GraphPad Prism. Quantitative data were expressed as mean ± standard deviation (SD). Differences between two groups were evaluated using Student's t-test. For comparisons among multiple groups, one-way ANOVA was used, followed by post-hoc tests to identify specific group differences. The level of significance was set at *, p < 0.05.

## Results

### LK-A suppressed the cell viability of OSCC cells

**Figure 1:**
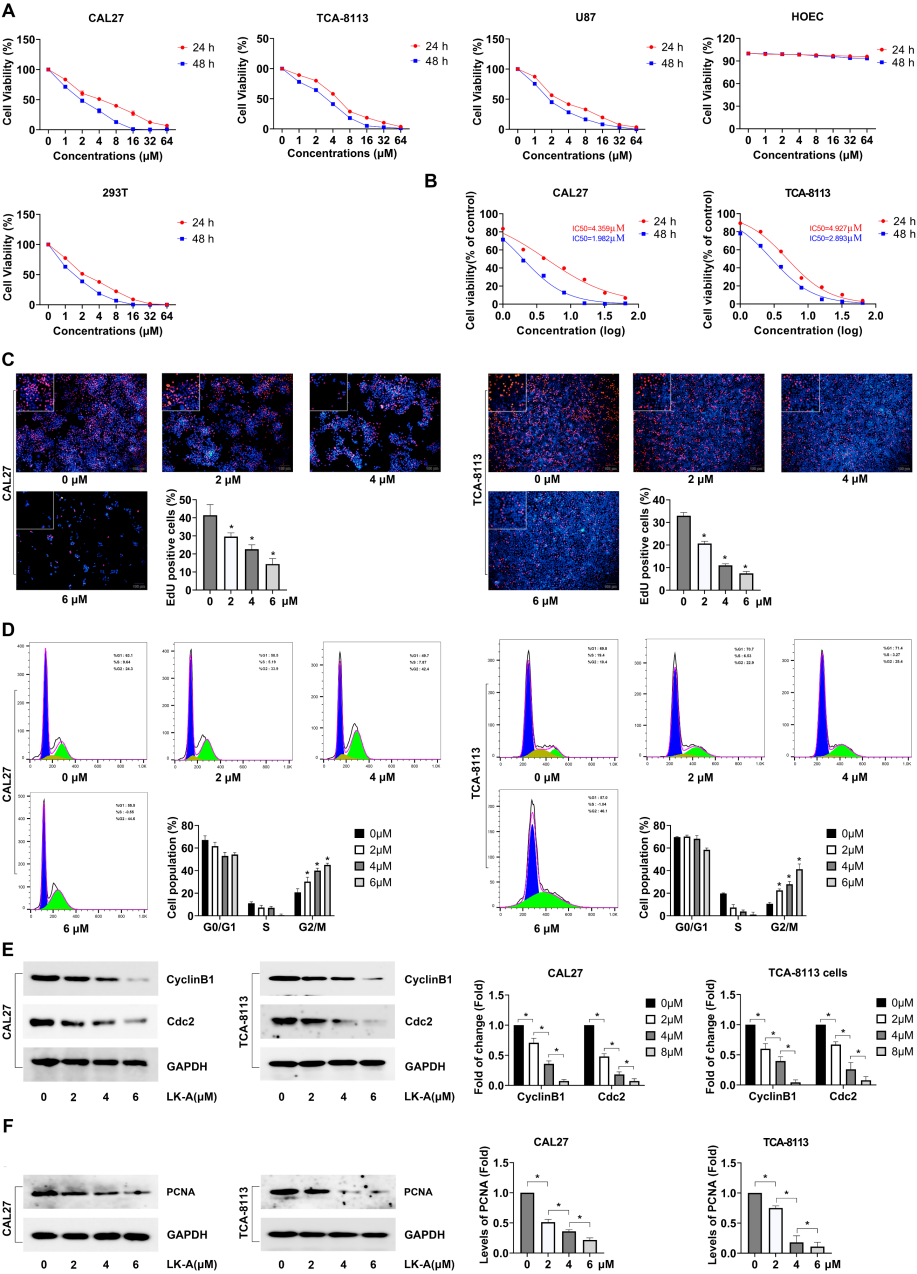
Impact of LK-A on the viability and proliferation of OSCC cell lines, U87, 293T and HOEC. (a) The viability of CAL27, TCA-8113, U87, 293T and HOEC.cells treated with varying concentrations of LK-A for 24 and 48 hours was assessed using CCK-8 assays. (b) The IC50 values of LK-A in CAL27 and TCA-8113 cells were calculated by GraphPad Prism 10.1.2. (c) EdU assay was conducted to determine CAL27 and TCA-8113 cell proliferation after treatment with varying concentrations of LK-A for 24 h.*P<0.05 versus 0μM LK-A-treated control group. (d) Cell cycle analysis was conducted to determine the cell cycle distribution of CAL27 and TCA-8113 cells after treatment with LK-A for 24 h. *P<0.05 versus 0μM LK-A-treated control group. (e) Western blot analysis was conducted to determine the expression of cell cycle-related proteins CyclinB1 and Cdc2 after treatment with 2,4,6 μM LK-A for 24 h. (f) Western blot analysis was conducted to determine the expression of PCNA protein after treatment with 2,4,6 μM LK-A for 24 h. *P<0.05 versus 0μM LK-A-treated control group.

Our study assessed the impact of LK-A on the viability of OSCC cell lines CAL27 and TCA-8113, utilizing CCK-8 and EdU assays to measure cell proliferation. Additionally, we evaluated the effects of LK-A on normal primary human oral mucosal epithelial cells (HOEC), as well as U87 and 293T cells. The results indicated a significant suppression of cell viability in OSCC cell lines, with LK-A exhibiting a dose-dependent effect. Notably, LK-A concentrations below 8 μM showed no significant inhibitory effect on HOEC (Figure 1A). Specifically, The IC50 values of LK-A were determined to be 4.36 μM for CAL27 cells and 4.93 μM for TCA-8113 cells at 24 h, and 1.98 μM for CAL27 cells and 2.89 μM for TCA-8113 cells at 48 h (Figure 1B). And the EdU assay demonstrated an decrease in the EdU positive rate as the dose of LK-A increased, suggesting an enhanced suppression of cell proliferation at higher concentrations (Figure 1C). Furthermore, cell cycle analysis indicated that LK-A induced a G2/M phase arrest in both CAL27 and TCA-8113 cells. This arrest is indicative of LK-A's ability to halt cell cycle progression, thereby reducing cell proliferation and contributing to the observed decrease in cell viability (Figure 1D). And Western blot analysis further confirmed increased dose of LK-A resulted in decreased expression of CyclinB1 and Cdc2 (Figure 1E). Moreover, we conducted PCNA detection and the results demonstrated a dose-dependent reduction in PCNA protein expression with increasing concentrations of LK-A, further confirming its inhibitory effect on cell proliferation (Figure 1E). These results collectively underscore LK-A's potential to significantly impair the proliferative capacity of OSCC cells in both CAL27 and TCA-8113 cell lines.

### LK-A suppressed the cell migration and invasion of OSCC cells

**Figure 2:**
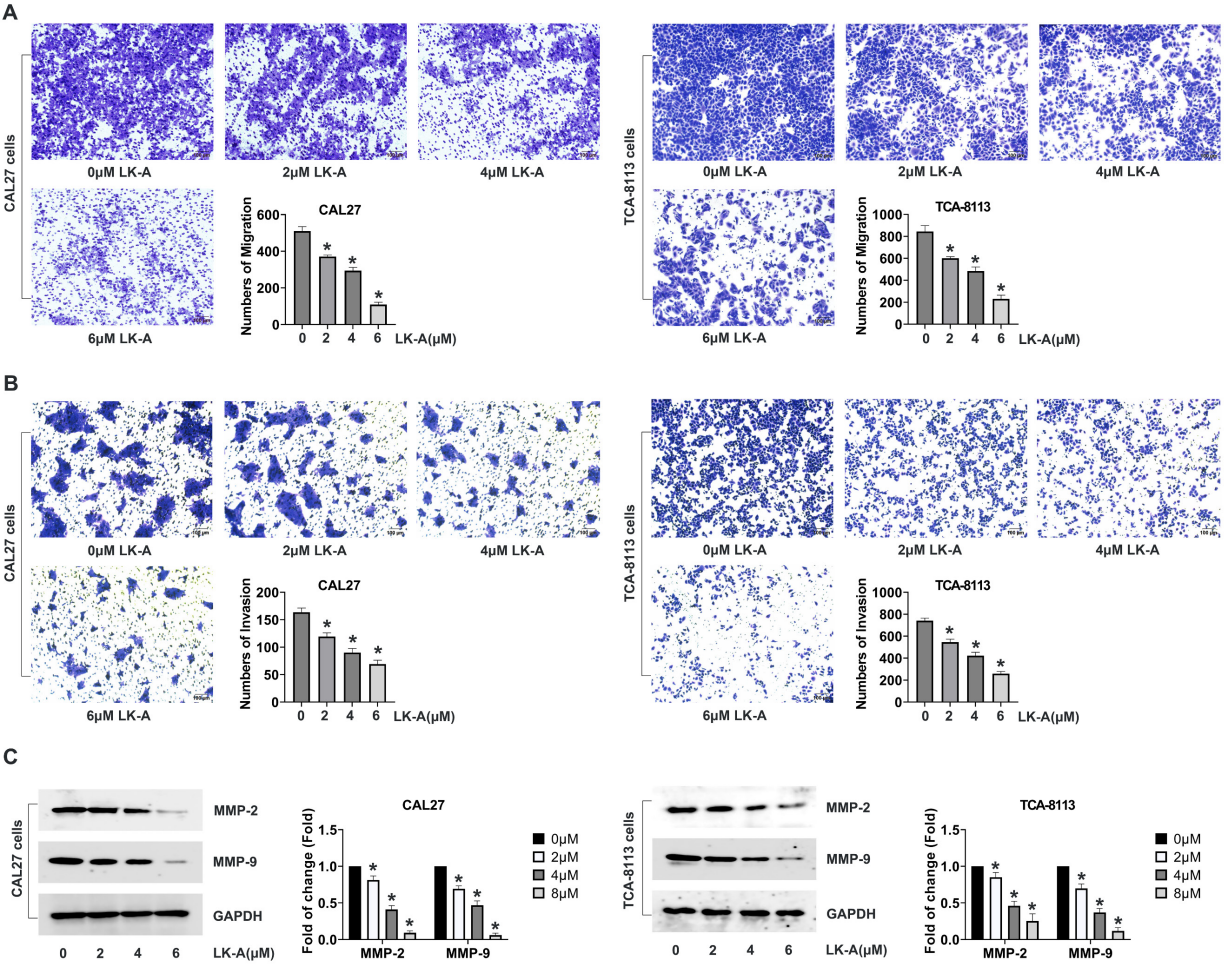
Impact of LK-A on the migration and invasion of OSCC cell lines CAL27 and TCA-8113. (a) Transwell migration assays were conducted to assess the effect of LK-A on cell mobility after treatment with LK-A for 24 h. (b) Invasion assays using Matrigel-coated membranes were conducted to assess the effect of LK-A on cell invasiveness after treatment with LK-A for 24 h. (c) Western blot analysis was performed to evaluate the expression of matrix metalloproteinases (MMP-2 and MMP-9) after LK-A treatment. *P<0.05 versus 0μM LK-A-treated control group.

Investigating the impact of LK-A on the migratory and invasive capabilities of OSCC cell lines CAL27 and TCA-8113, we utilized transwell assays, with and without Matrigel coating, to assess cell migration and invasion, respectively. The migration assay revealed a significant decrease in the number of cells migrating through the porous membrane in the presence of LK-A, indicating its strong inhibitory effect on cell mobility (Figure 2A). Similarly, the invasion assay, employing a Matrigel-coated membrane to mimic the extracellular matrix, showed that LK-A substantially reduced the invasive potential of both cell lines compared to control groups (Figure 2B).

Further analysis was conducted to evaluate the expression of matrix metalloproteinases (MMP-2 and MMP-9), enzymes critical for cancer cell invasion and metastasis 16. Western blot assays demonstrated that LK-A treatment led to a marked downregulation of MMP-2 and MMP-9 expression in both CAL27 and TCA-8113 cells (Figure 2C). This reduction in MMP levels correlates with the observed decrease in cell migration and invasion, supporting the notion that LK-A exerts its anti-metastatic effects, at least in part, by modulating the expression of these matrix-degrading enzymes.

### LK-A promoted apoptosis in OSCC cells

**Figure 3:**
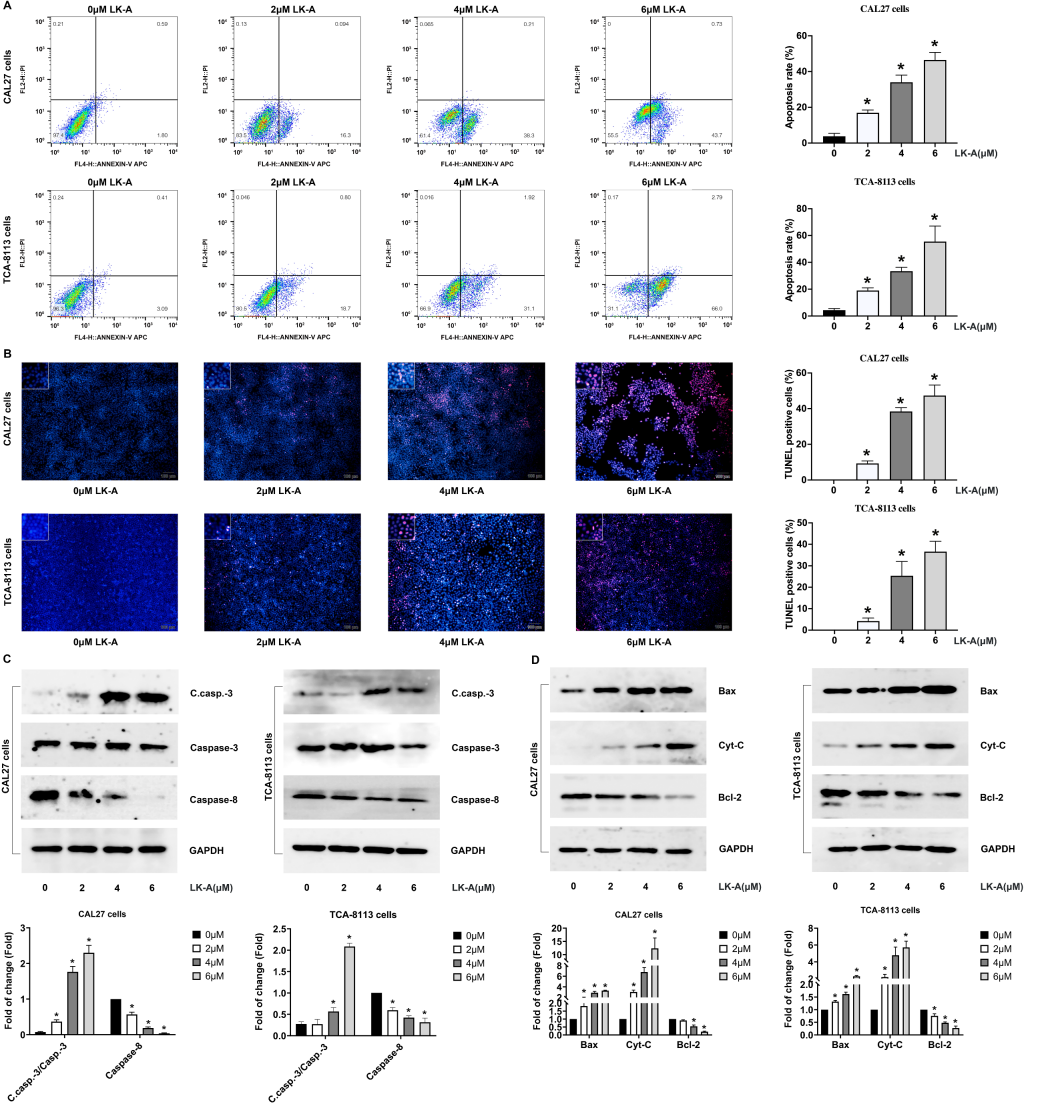
Impact of LK-A on apoptosis in OSCC cell lines CAL27 and TCA-8113. (a) Annexin V-FITC/PI staining was conducted to assess the apoptotic effects of LK-A on CAL27 and TCA-8113 cells after treatment with varying concentrations of LK-A for 24 h.*P<0.05 versus 0μM LK-A-treated control group. (b)TUNEL assay was performed to detect DNA fragmentation, showing a higher number of TUNEL-positive cells after LK-A treatment, indicating increased apoptosis. *P<0.05 versus 0μM LK-A-treated control group. (c) Western blot analysis of apoptosis-related proteins demonstrated a dose-dependent increase in cleaved caspase-3 (C.casp.-3) and a decrease in caspase-8 levels in LK-A-treated cells. *P<0.05 versus 0μM LK-A-treated control group. (d) Western blot analysis showed enhanced expression of pro-apoptotic factors Bax and Cyt-C, and decreased levels of the anti-apoptotic protein Bcl-2 in cells treated with LK-A.*P<0.05 versus 0μM LK-A-treated control group.

We further evaluated the pro-apoptotic effects of LK-A on OSCC cell lines by conducting Annexin V-FITC/PI staining and TUNEL assays. These analyses confirmed that LK-A significantly increased apoptosis in OSCC cells. Annexin V-FITC/PI staining revealed a higher percentage of apoptotic cells in the LK-A-treated groups compared to controls, indicating its potent apoptotic induction capability (Figure 3A). Similarly, the TUNEL assay, which detects DNA fragmentation, corroborated the increased apoptotic rate, showing a higher number of TUNEL-positive cells after LK-A treatment (Figure 3B).

To elucidate the molecular mechanisms underlying these effects, Western blot analysis was performed. The results indicated that LK-A treatment led to a dose-dependent increase in the levels of apoptosis-related C.casp.-3 and a decreased caspase-8 (Figure 3C). Moreover, the expression of pro-apoptotic factors, Bax and Cyt-C, was notably enhanced, while the level of the anti-apoptotic protein Bcl-2 was concurrently decreased in cells treated with LK-A (Figure 3D). These molecular changes confirm the activation of the apoptotic pathway by LK-A in OSCC cells.

### LK-A suppresses PI3K/AKT signaling in OSCC Cells, with effects reversed by AKT activation

**Figure 4:**
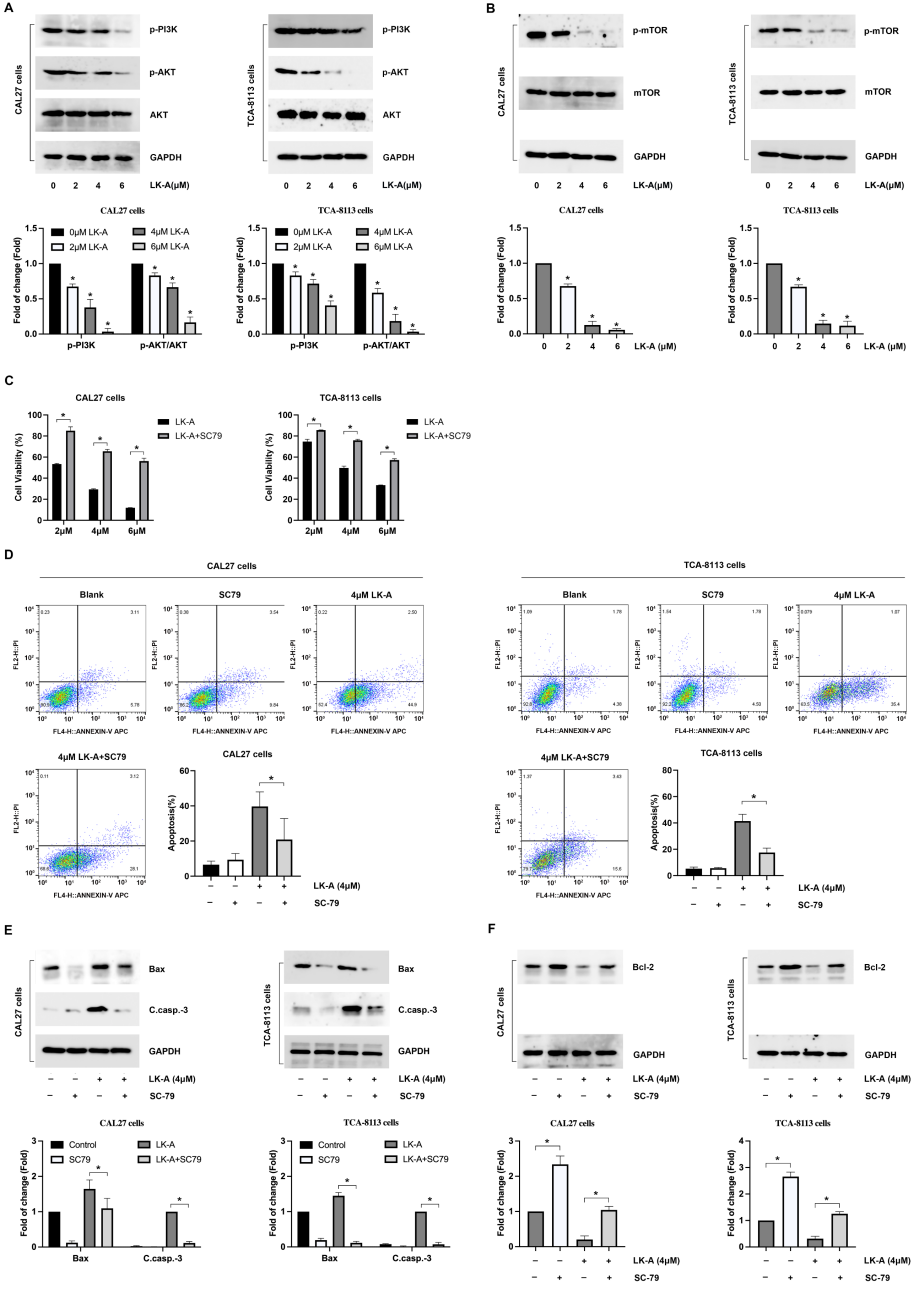
Effects of LK-A on the PI3K/AKT signaling pathway in OSCC cell lines CAL27 and TCA-8113. (a) Western blot analysis showed reduced levels of p-PI3K and p-AKT following LK-A treatment, indicating suppression of the PI3K/AKT pathway. *P<0.05 versus 0μM LK-A-treated control group. (b) Western blot analysis of mTOR, a downstream effector of PI3K/AKT signaling, revealed decreased p-mTOR expression levels after LK-A treatment. *P<0.05 versus 0μM LK-A-treated control group. (c) CCK-8 assays were conducted to demonstrate that the LK-A-induced suppression of OSCC cell proliferation was reversed by the AKT activator SC79. (d) Flow cytometry analysis indicated that the increase in apoptotic cell percentage induced by LK-A was attenuated when cells were co-treated with SC79. (e) Western blot analysis showed that LK-A-induced upregulation of pro-apoptotic proteins Bax and cleaved caspase-3 was mitigated by SC79 treatment. (f) Western blot analysis revealed that the reduction in the anti-apoptotic protein Bcl-2, induced by LK-A, was countered by SC79 administration, leading to increased Bcl-2 levels.

To understand the mechanisms by which LK-A exerts its antitumor effects, we found a significant reduction in AKT phosphorylation (p-AKT), indicating the inhibition of this critical signaling pathway. Western blot analysis revealed diminished levels of p-PI3K, p-AKT (Figure 4A) and associated downstream effectors, such as mTOR (Figure 4B), highlighting the pathway's suppression following LK-A treatment. This suppression correlated with the observed decrease in cell proliferation and viability, suggesting that the antitumor activity of LK-A is mediated through PI3K/AKT pathway inhibition.

To further elucidate the role of AKT signaling in LK-A's mechanism, we utilized the AKT activator SC79. Upon SC79 treatment, the LK-A-induced suppression of OSCC cell proliferation was reversed, as evidenced by CCK-8 assays (Figure 4C), indicating the pivotal role of the PI3K/AKT pathway in mediating the compound's effects.

Additionally, flow cytometry analyses showed that the increase in apoptotic cell percentage induced by LK-A was attenuated when OSCC cells were co-treated with SC79 (Figure 4D). Molecularly, LK-A-induced upregulation of pro-apoptotic proteins Bax and cleaved caspase-3 was mitigated by SC79 (Figure 4E). Conversely, the reduction in the anti-apoptotic protein Bcl-2, seen with LK-A treatment, was countered by SC79 administration, leading to an increase in Bcl-2 levels (Figure 4F). These integrated findings demonstrate that LK-A's antiproliferative and pro-apoptotic actions in OSCC cells are significantly driven by the suppression of the PI3K/AKT signaling pathway.

### LK-A Suppressed Tumor Growth in Mouse Xenograft Models

**Figure 5:**
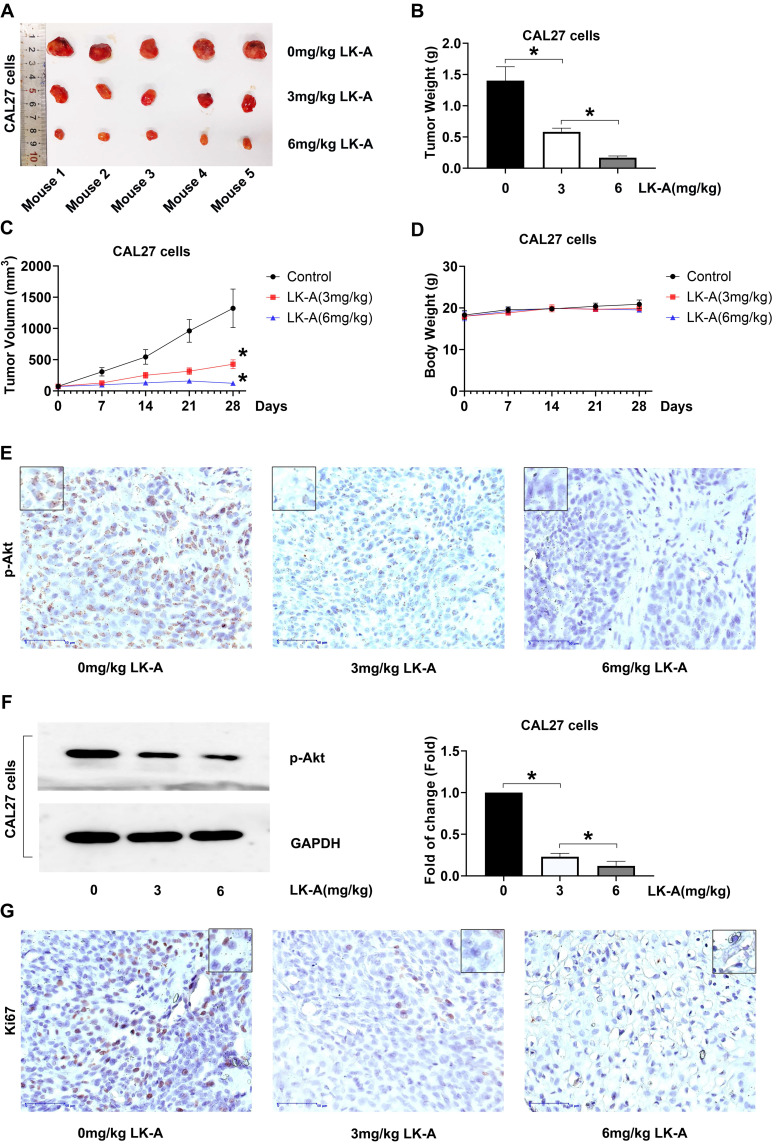
LK-A suppresses tumor growth in mouse xenograft models of OSCC. (a) Tumor growth in nude mice subcutaneously inoculated with CAL27 cells was significantly reduced by LK-A treatment at doses of 3 and 6 mg/kg administered intraperitoneally every three days. (b) Tumor weights in LK-A-treated mice were notably lower than those in the negative control group. (c) Mean tumor volumes in LK-A-treated mice at 3 and 6 mg/kg were significantly less than those in the negative control group. *P<0.05 versus 0mg/kg LK-A-treated control group. (d) Body weights of the experimental animals showed no significant loss during LK-A treatment. (e) IHC analysis revealed a marked inhibition of p-Akt expression in tumor tissues of LK-A-treated mice. (f) Western blot analysis confirmed reduced p-Akt levels in LK-A-treated tumor tissues. (g) IHC staining for Ki-67 showed decreased expression in LK-A-treated tumors, indicating reduced cell proliferation.

In our *in vivo* assessment, CAL27 cells were subcutaneously inoculated into nude mice to establish tumor xenografts. The mice were treated with LK-A at doses of 3 and 6 mg/kg intraperitoneally every three days. LK-A significantly reduced tumor growth (Figure 5A), with the tumor weights in LK-A-treated mice being notably lower than those in the negative control group (Figure 5B). The mean tumor volume for LK-A-treated mice at 3 and 6 mg/kg treatment were also less than that of the negative control group (Figure 5C). Notably, these therapeutic effects were achieved without significant loss in body weight of the experimental animals (Figure 5D).

Immunohistochemistry (IHC) and Western blot assays conducted on the tumor tissues further revealed that LK-A treatment led to a marked inhibition of p-Akt expression. The reduced p-Akt levels, confirmed by both IHC (Figure 5E) and Western blot (Figure 5F) analyses, indicate the effective suppression of the PI3K/AKT signaling pathway in the tumor xenografts treated with LK-A. Additionally, the immunohistochemical staining for Ki-67 showed decreased expression in the LK-A-treated tumors, suggesting a reduction in cell proliferation (Figure 5G). These comprehensive results demonstrate that LK-A effectively inhibits tumor growth *in vivo* in mouse xenograft models of OSCC, with a concurrent decrease in p-Akt expression, highlighting its potential therapeutic efficacy against OSCC through the suppression of the PI3K/AKT pathway.

### LK-A inhibited cell proliferation in a patientderived tumor xenograft model

**Figure 6:**
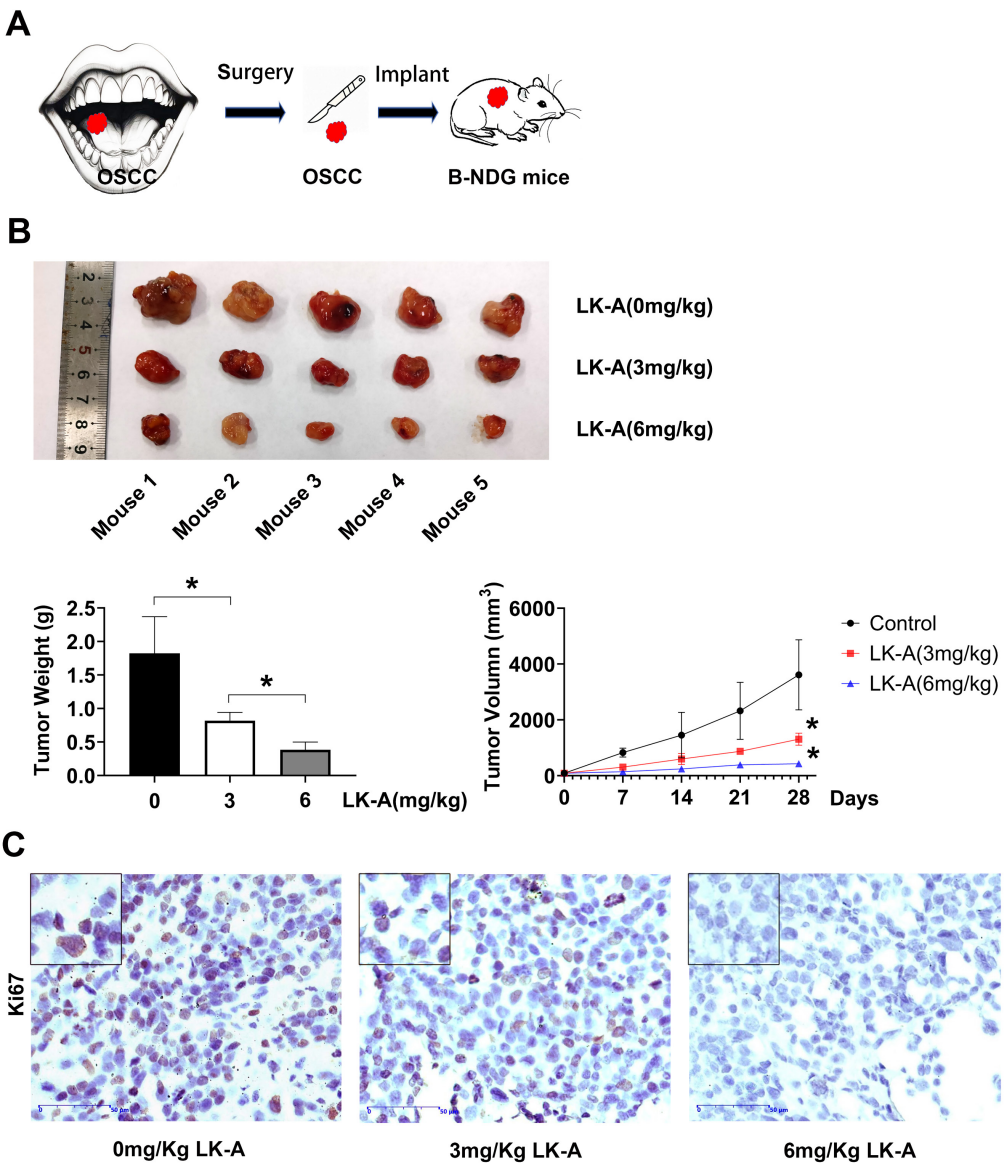
*In vivo* anti-tumor activity of LK-A in the PDTX tumor model. (A)A diagram depicting the workflow for developing a patient-derived tumor model.(B) Mean tumor weight and tumor growth trajectories in mice following treatment with 0 mg/kg, 3 mg/kg, or 6 mg/kg of LK-A. (C) Representative Ki67 immunohistochemical-labeled tumor tissue samples from various treatment groups. Data are presented as mean ± SD (n = 5).

In recent years, patient-derived tumor xenograft (PDTX) models have been increasingly used to better replicate human tumors, offering greater clinical relevance compared to traditional cell line-based xenograft models. In this study, we conducted an *in vivo* experiment using a PDTX glioma model, where human tumor samples from a patient were implanted into B-NDG mice (Figure 6A). Treatment with LK-A was well-tolerated and led to significant tumor regression in immunocompromised mice bearing patient-derived OSCC tumors (Figures 6B). To further validate the efficacy of LK-A in inhibiting OSCC growth, we performed an *ex vivo* immunohistochemistry (IHC) staining assay (Figures 6C). The results showed a significant reduction in Ki67-positive cells in tumors treated with 6 mg/kg of LK-A compared to the control group, suggesting that the inhibition of tumor cell proliferation may contribute to LK-A's anti-tumor effects.

## Discussion

This study delineated the anti-cancer effects of LK-A on OSCC, with a particular focus on its modulation of the PI3K/Akt signaling pathway. Our results demonstrated that LK-A significantly inhibited cell proliferation, migration, and invasion, and induced apoptosis in OSCC cell lines. These effects were corroborated *in vivo*, where LK-A treatment led to a notable reduction in tumor growth in xenograft models, underscoring its potential therapeutic efficacy.

The suppression of the PI3K/Akt pathway by LK-A, evidenced by the decreased phosphorylation of Akt and subsequent downregulation of downstream effectors like mTOR 17, highlights the pathway's critical role in OSCC pathophysiology. Martins *et al.* confirmed higher p-AKT and p-mTOR expression during carcinogenesis of oral mucosa in OSCC specimens 18. This upregulation is associated with the progression and poor prognosis of OSCC 19,20. AKT, as a central component of this pathway, is often found to be overactive, promoting cell proliferation and inhibiting apoptosis 21. Similarly, mTOR, a downstream target of AKT, is also commonly overexpressed or hyperactivated in OSCC, contributing to tumor development and progression 22. Inhibiting the PI3K/AKT/mTOR pathway has shown promise in reducing OSCC tumor growth and improving sensitivity to treatment 23. The ability of LK-A to inhibit this pathway suggests a mechanism that contributes to its anti-tumorigenic effects, given the central role of PI3K/Akt signaling in cell survival, growth, and metastasis. The reversal of LK-A's anti-proliferative effects by the Akt activator SC79 further confirms the specificity of LK-A's action on the PI3K/Akt pathway.

Moreover, we observed that LK-A treatment led to a notable decrease in the expression of MMP2 and MMP9 in OSCC cells. Considering the critical role of these matrix metalloproteinases in degrading the extracellular matrix, a key process in cancer cell invasion and metastasis, this reduction is significant 24. Our findings that LK-A can inhibit the invasive and migratory abilities of OSCC cells *in vitro* further underscore its potential as an anti-metastatic agent. The downregulation of MMP2 and MMP9 by LK-A suggests a direct impact on the cells' ability to invade surrounding tissues and form distant metastases, which are crucial steps in the progression of OSCC and are directly correlated with patient prognosis.

The relationship between MMP expression and OSCC invasiveness is well-established in the literature, with higher levels of these enzymes often found in more aggressive and metastatic tumors 25,26. By impairing the MMP-dependent breakdown of the extracellular matrix, LK-A may effectively hinder the invasive process, potentially limiting the spread of OSCC cells within the body. This is particularly relevant for patient outcomes, as the extent of local invasion and the presence of metastases are major determinants of survival in OSCC 27,28. The ability of LK-A to modulate the expression of these proteases highlights its potential as a therapeutic agent that not only targets primary tumor growth but also mitigates the risk of dissemination and establishment of secondary tumors.

The observed *in vivo* efficacy of LK-A in reducing tumor volumes is a pivotal finding, underscoring its potential as an anti-cancer agent for oral squamous cell carcinoma (OSCC). This reduction in tumor size, coupled with the downregulation of key proliferation and signaling pathway markers like Ki-67 and p-Akt, points to the direct impact of LK-A on cellular processes critical for tumor growth and survival. Ki-67, a well-known marker for cell proliferation, is commonly used to gauge the growth rate of tumors, with higher levels indicating rapid cell division 29. The decrease in Ki-67 expression following LK-A treatment suggests a slowdown in the proliferation rate of OSCC cells. Similarly, the reduction in p-Akt levels indicates the inhibition of the PI3K/Akt signaling pathway, a crucial route that promotes cell survival, growth, and proliferation in many cancers, including OSCC 30. By targeting this pathway, LK-A disrupts a key molecular mechanism that cancer cells exploit for their uncontrolled growth and resistance to cell death. Importantly, the lack of significant toxicity, as evidenced by the absence of substantial weight loss in the treated mice, highlights LK-A's favorable safety profile.

## Conclusions

In conclusion, LK-A emerges as a potent inhibitor of the PI3K/Akt pathway, with significant anti-tumorigenic effects in OSCC. This work advances our understanding of LK-A's mechanistic actions and supports the potential of PI3K/Akt pathway inhibitors in OSCC treatment.
